# A Depth-Adjustment Deployment Algorithm Based on Two-Dimensional Convex Hull and Spanning Tree for Underwater Wireless Sensor Networks

**DOI:** 10.3390/s16071087

**Published:** 2016-07-14

**Authors:** Peng Jiang, Shuai Liu, Jun Liu, Feng Wu, Le Zhang

**Affiliations:** College of Automation, Hangzhou Dianzi University, Hangzhou 310018, China; liushuaihdu@163.com (S.L.); liujunhdu@163.com (J.L.); fengwu@hdu.edu.cn (F.W.); lezhang@hdu.edu.cn (L.Z.)

**Keywords:** underwater wireless sensor networks (UWSNs), two-dimensional convex hull, spanning tree, time marker, network reliability, full network connectivity

## Abstract

Most of the existing node depth-adjustment deployment algorithms for underwater wireless sensor networks (UWSNs) just consider how to optimize network coverage and connectivity rate. However, these literatures don’t discuss full network connectivity, while optimization of network energy efficiency and network reliability are vital topics for UWSN deployment. Therefore, in this study, a depth-adjustment deployment algorithm based on two-dimensional (2D) convex hull and spanning tree (NDACS) for UWSNs is proposed. First, the proposed algorithm uses the geometric characteristics of a 2D convex hull and empty circle to find the optimal location of a sleep node and activate it, minimizes the network coverage overlaps of the 2D plane, and then increases the coverage rate until the first layer coverage threshold is reached. Second, the sink node acts as a root node of all active nodes on the 2D convex hull and then forms a small spanning tree gradually. Finally, the depth-adjustment strategy based on time marker is used to achieve the three-dimensional overall network deployment. Compared with existing depth-adjustment deployment algorithms, the simulation results show that the NDACS algorithm can maintain full network connectivity with high network coverage rate, as well as improved network average node degree, thus increasing network reliability.

## 1. Introduction

Underwater wireless sensor networks (UWSNs) are an aquatic environment monitoring network system that consists of many nodes with data acquisition, storage, processing, and wireless acoustic transmission features. UWSNs have extensive applications in water environmental pollution prediction, ocean exploitation, marine monitoring system, and other areas [1,2,3,4]. With the increasing depletion of land resources, coastal countries are paying increasing attention to maritime rights and resource exploitation, and the ocean economy plays a vital role in the development of the world economy. Therefore, UWSN technology has become a popular research topic in the field of wireless sensor networks. As a technology extension of terrestrial wireless sensor networks (TWSNs) [5,6] to aquatic environments, UWSNs inherit some characteristics of TWSNs, however due to the special application environment and underwater acoustic communication model of UWSNs, they ordinarily bring some new characteristics, namely, their network structure, which is distributed in three dimensions, high latency of acoustic signal, limited bandwidth of communication, high transmission bit-error-rates, constrained movement of nodes, very limited energy, and others [1,7,8]. In view of the abovementioned characteristics of UWSNs, the deployment algorithms and routing protocols of TWSNs cannot be directly migrated to the UWSNs. These new characteristics must be combined to design more reasonably effective deployment algorithms and positioning and routing protocols for UWSNs [9,10,11]. The protocol and algorithm design of UWSNs mostly relate to node deployment, topology control, node localization, time synchronization, routing protocol, and others. Meanwhile, the node deployment is not only directly related to the quality of network monitoring but also dramatically affects subsequent processing on the design of all kinds of protocols and algorithms. Therefore, in this study, this basic and crucial problem is further investigated, namely, node deployment.

According to whether the target location of the node is determined, the node deployment algorithm of UWSNs can be divided into the following two major categories: stochastic deployment algorithm and deterministic deployment algorithm [12]. After all of the nodes are scattered on the monitoring water surface uniformly and randomly by boat or aircraft, if these nodes only execute random depth adjustment in the vertical direction, then the deployment strategy is referred to as stochastic deployment. If the node can be moved to a definite position calculated by the corresponding optimization algorithm, then the deployment algorithm is referred to as deterministic deployment. Compared to the stochastic deployment, the deterministic deployment can achieve better network performance and therefore is widely used. However, for the node deterministic deployment, based on the assumption of node mobility, the existing deterministic deployment algorithms can be classified into three categories, namely, vertical movement deployment [13,14,15], static deployment [16,17,18], and free movement deployment [19,20,21].

In this study, we mostly consider vertical movement deployment (depth-adjustment deployment). Depth-adjustment deployment refers to the deployment in which all of the nodes only execute depth-adjustment along the vertical direction in the underwater monitoring space. Different from free movement deployment, vertical movement deployment can only achieve node vertical depth adjustment by adjusting the length of the rope or some other economical method [13], thus the vertical movement deployment strategy or algorithm has strong feasibility and applicability. Among existing vertical movement deployments, the deployment strategy based on graph theory in the underwater node deployment is widely used. This algorithm adopts relevant concepts (graph coloring problem or connected dominating set) of graph theory to adjust the depth of nodes through the depth-adjustment to reduce redundant coverage, thus completing the overall 3D coverage. Akkaya et al. [14] proposed a self-deployment depth adjustment algorithm that mainly focuses on maximizing network coverage by constantly adjusting node depths to reduce coverage overlaps between two neighboring nodes. Meanwhile, this algorithm adopts graph coloring problem to solve the distribution of network group IDs, based on coverage overlaps of intra-cluster nodes for different node assigned with different group IDs, and the cluster head node applies the centralized approach to nodes executing depth adjustment, decreasing coverage overlaps and thus increasing overall network coverage for the 3D monitoring space. Senel et al. [15] proposed depth adjustment algorithms based on a connected dominating set, and this algorithm is derived from [14]. Compared with the latter algorithm, the former algorithm initially constitutes a connected backbone, and then the dominant node respectively optimizes its non-dominators located inside their connected dominating set in an iterative way. Moreover, the algorithm ensures the full network connectivity, maximizes network overall coverage rate, and reduces the deployment energy consumption.

Meanwhile, many scholars have proposed deployment algorithms based on computational geometry for traditional sensor networks and supported many applications in real industrial markets. Heo et al. [22] proposed a self-deployment algorithm based on Voronoi diagrams. The algorithm constant constructs Voronoi diagrams of all nodes to reflect the nodes’ movement state and optimize the location of nodes, thus improving network operator performance. Wang et al. [23] used Voronoi diagrams to detect network coverage holes and to control the movement of nodes to repair coverage holes. The algorithm argues that calculating the correct Voronoi polygons can help to optimize its healing efficiency of coverage holes, if they exist. However, network connectivity is not considered. The construction of the Voronio diagram needs to execute global computation, so the communication costs are large. In recent years, some scholars have transferred the concept of computational geometry deployment strategies to UWSN applications. Wu et al. [24] proposed Voronoi-based depth-adjustment algorithm (VBDA) for UWSNs. First, all of the nodes are randomly deployed on the water surface through the formation of the Voronoi polygon of each node to calculate the network coverage redundancy, the Voronoi region area of each node in same layer to determine the depth-adjustment distance, and then the iterative operating process of depth adjustment until the last layer network deployment arrives at the water bottom threshold. The VBDA is first proposed based on the Vonoroi deployment strategy for UWSNs. Through the vertical depth adjustment of the nodes to reduce the network coverage redundancy, the 3D network coverage is increased. However, the algorithm does not consider the network connectivity between two neighboring layers, which makes the network connectivity performance very poor with a small *α* (the ratio of node communication radius to sensing radius). Moreover, as the process of network deployment proceeds, fewer nodes are available to execute network deployment when the process of depth-adjustment reaches the water bottom threshold, then its network coverage performance is relatively inferior. Meantime, the VBDA is a centralized algorithm, and all nodes have more communication costs and then consume more communication energy, thereby affecting network operator performance.

The deployment issues mentioned above, considering the above-proposed deployment algorithms of UWSNs based on computational geometry consisting of vertical movement deployment strategy, the VBDA cannot improve network coverage performance while ensuring full network connectivity. Furthermore, the communication energy consumption still has ample room for improvement. Therefore, we proposed a node depth adjustment deployment algorithm based on two-dimensional (2D) convex hull and spanning tree (NDACS). A sink node is fixed at the center of the monitored water surface, and all of the nodes are scattered on the monitored water surface uniformly and randomly and are in a sleep state. First, the sink node sends control information to activate three nodes according to the node ID from small to large and forms a very simple 2D convex hull (triangle). Thereafter, it uses the geometric characteristics of the 2D convex hull and empty circle to minimize the 2D plane network coverage overlaps as its optimization goal. The optimal sleep node that needs be activated is found, whereas the 2D coveragence efficiency could be greatly improved. When the coverage threshold of the first layer is reached, the algorithm forms the best fully 2D node activation convex hull. At the same time, most of the nodes are in a sleep state to save overall network energy. Thereafter, the sink node acts as a root node of all active nodes on the 2D convex hull and gradually forms a small spanning tree. Eventually, a new spanning forest is formed through the adjustable communication radius model in the 2D water surface. The child nodes closer to the sink node have greater probability of data transmission and relay and have to consume more energy. Therefore, for the network to be able to continuously operate stably, more nodes must be deployed near the sink node, in which more subtrees are found around the sink node, and the size of surrounding subtrees should be smaller. We adopt the time marker to define the 2D network level and make the interlayer neighbor intersection be as close to the parent node of the spanning tree as possible, increasing the node deployment density around the sink node and balancing network energy consumption. Finally, the active node is used as the parent node of its neighbor sleep node, and the depth-adjustment strategy based on the time marker is used to achieve the 3D overall network coverage. The simulation results show that compared to the typical depth-adjustment deployment algorithm (VBDA), the NDACS algorithm can maintain full network connectivity with a high network coverage rate while reducing communication energy consumption with better network reliability during deployment. Compared with existing depth-adjustment deployment algorithms, our proposed algorithm contributes through the following aspects:

(1) The geometric characteristics of the 2D convex hull and empty circle is used to minimize the 2D plane network coverage overlaps, with a minimum number of activated nodes to meet the requirement of the first layer of the network deployment.
(2) The activation scheduling mechanism is adopted to reduce the energy consumption of communication between nodes.
(3) The adjustable communication radius model is established to ensure that all active nodes have full network connectivity in the first layer. In addition, in the process of depth adjustment, the communication connection is guaranteed between the parent and its child nodes in real time.
(4) Depth adjustment is based on the time marker, adjacent time marker neighbor nodes set is dominated by its parent node, and it is a perfect approach to balance the density of node deployment. When the set is empty, the neighbor nodes intersect to execute depth-adjustment assignment, ensuring that the network deployment performance of near water bottom is relatively good and that network operation is more reliable. Overall, our work optimizes network coverage rate under the premise of full network connectivity, reducing the energy consumption of communication.

The rest of this paper is organized as follows: in Section 2, the related works about the node deployment problem in three categories algorithm for UWSNs. In Section 3, the models of UWSNs and related definitions are described. In Section 4, the problem is analyzed, and the details of the NDACS algorithm are implemented. In Section 5, the communication complexity and time complexity of network deployment for NDACS algorithm are analyzed. In Section 6, the description of algorithm simulation and the detailed analysis of the simulation results are discussed. Finally, in Section 7, the conclusions and future work of our study are drawn.

## 2. Related Works

In addition to a number of different strategies for node deployment of UWSNs, many researchers have focused on static deployment [16,17,18] and free movement deployment [19,20,21]. The static deployment algorithm refers to the assumption that nodes do not have the ability to move. The nodes need artificial deployment to a fixed location determined by advance calculation, and when the network deployment is completed, besides, the nodes no longer move. Alam et al. [16] used different polyhedrons to fill three-dimensional (3D) deployment space and study the maximization of 3D coverage space with a minimum number of nodes, offering mathematical proof and derivation to explain why truncated octahedral cells result in the best strategy and achieve the greatest network coverage performance. Subsequently, basing on the study of [16], Alam et al. [17] used space-filling truncated polyhedrons to execute network coverage deployment by controlling the cell-vertex nodes’ active or sleep states, and adjusting the space-filling polyhedron radius can be flexible to achieve *k*-coverage. Liu et al. [18] proposed a geometric hierarchical clustered deployment strategy where the cluster head nodes are alternated by using the geometric characteristics of the cluster shape and constraint condition that ensures network connectivity and full coverage. The optimal network lifetime mathematical model and cluster shape model are derived, and optimal node deployment is achieved. On the whole, the node goal location has been determined before the real deployment. Therefore, the static deployment algorithm usually can achieve good network coverage or connectivity performance. However, the node needs to be fixed to the definite position through artificial means, and obviously, the difficulty of deployment is excessively large and the deployment cost is excessively high for aquatic environments. In addition, when the network scale is expanded or topological changes occur because of the effect of water environment (water flow or animal collision), the applicability of the static deployment algorithm decreases rapidly.

Free movement deployment refers to the assumption that nodes have the ability to move freely in each direction and can be moved to any location in the 3D network space. Considering the covering of the uneven isolated incident in the monitoring area, Xiana et al. [19] first proposed fish swarm-inspired node deployment and subsequently [20] proposed a similar algorithm—the particle swarm-inspired node deployment. These two algorithms simulate the behavior of fish swarms or particle swarms and introduce the model of congestion degree control. The proposed algorithms can drive nodes to cover the events and match event distribution density in the water environment. The author only considers the coverage rate of the events and ignores the network connectivity rate during the deployment process. Li et al. [21] proposed the 3D virtual forces deployment algorithm. This algorithm uses virtual force to adjust node location and improve network coverage rates and connectivity rates. However, this algorithm could not achieve full network connectivity, that is, the network connectivity rate is 1. For this kind of free movement, in all of the abovementioned proposed deployment algorithms, the node has the ability to move in each direction and can move to an optimized location where network coverage and connectivity performance are obviously improved. However, free movement deployment needs to equip these nodes with autonomous underwater vehicle (AUV) or other mobile devices under normal circumstances then its strategy induces relatively high production costs, which make the node free movement deployment algorithm difficult to spread in the practical UWSNs application.

As a research team which has remained focused on WSNs, our team also proposed some node deployment algorithms for UWSNs in recent years [25,26,27,28]. The node self-deployment algorithm based on an uneven cluster with radius adjusting (URSA) for UWSNs was proposed in [25], and the difference between the URSA and NDACS was the core concept of the URSA algorithm based on uneven clustering. However, our proposed algorithm main is based on computational geometry. The algorithms in [25,26] belong to depth-adjustment deployment, while the algorithms in [27,28] belong to free movement deployment.

## 3. Network Models and Related Definitions

### 3.1. Network Models

#### 3.1.1. Network System Model

**Figure 1:**
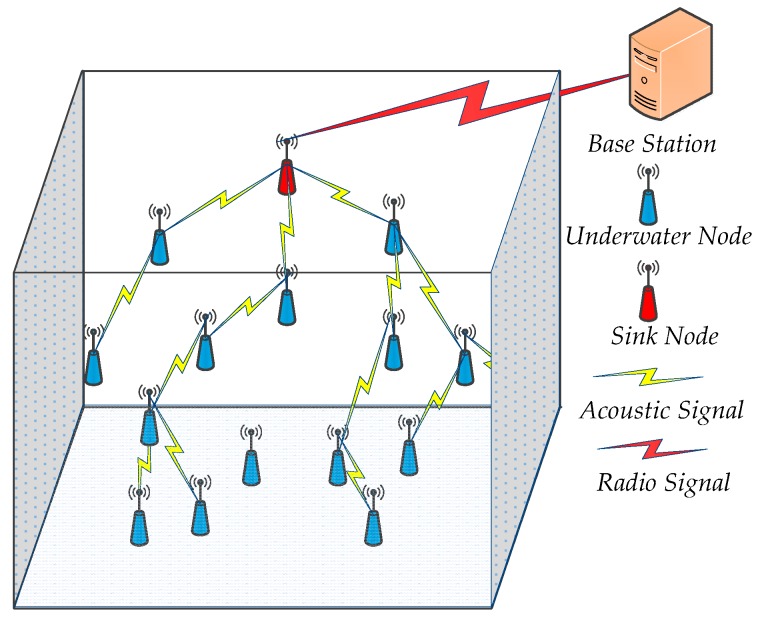
3D UWSNs system model.

Referring to the 3D characteristic of UWSNs in this paper, we assume that *n* nodes are distributed randomly and uniformly on the water surface, that they are floating with the help of buoys, and that the area of 3D monitoring space is *L × W × H*. A sink node is fixed at the center of monitoring water surface. The typical UWSNs model is described in Figure 1, including the following preliminaries:

(1) The node has no ability to perceive global coordinate information in 3D network space, and merely the node perceives own depth information.
(2) Each node has a unique identity ID.
(3) The boundary effect is negligible because the node communication radius *Rc* and sensing radius *Rs* are significantly smaller than the length *L*, width *W*, or height *H* of the 3D monitoring space.
(4) All nodes use a uniform acoustic signal to transmit and receive information, and the sink node transmits the information to the base station by radio signal.
(5) All nodes are identical and have the same initial energy *E_init_*, sensing radius *Rs*, and the initial communication radius *Rc_init_*. Moreover, the energy consumption of the sink node is negligible.

#### 3.1.2. Node Perception Model

The Boolean perception model describes the node sensing in this study. The coordinate of node *S_i_* is (*x_i_, y_i_, z_i_*), and the Euclidean distance between *S_i_* and point *p*(*x*, *y*, *z*) is $d(s_{i},p)=\sqrt{{\left(x-x_{i}\right)}^{2}+{\left(y-y_{i}\right)}^{2}+{\left(z-z_{i}\right)}^{2}}$. Therefore, under this perception model, the probability of point *p* is perceived by node *S_i_* and can be presented as follows:

(1) $$c_{p}\left(S_{i}\right)=\left\{ \begin{array}{l} 1,\;d\left(S_{i},p\right)<Rs\\ 0,\;d\left(S_{i},p\right)\geq Rs \end{array}\right.$$

where *Rs* denotes the sensing radius of node *S_i_*.

#### 3.1.3. Adjustable Communication Radius Model

The maximum communication radius of the node is *Rc*, and its initial value is *Rc_init_*, the adjustable level of communication radius is defined as *θ* and has an initial value of 1. Therefore, we can obtain the relationship between *Rc* and *θ* through the following formula:

(2) $$Rc=Rc_{init}+\left(1+\theta \right)B_{u}$$

where *B_u_* is the increasing communication range per communication level.

#### 3.1.4. Node Energy Consumption Model

With the energy consumption of node sensing and data receiving significantly smaller than that of data transmission and node movement [29], so we only consider the latter as a primary component of energy consumption. In this study, the energy consumption model of the node based on the method is mentioned in [30,31], and it can be calculated through the following formula:

(3) $$E_{c}(d,f)=P_{0}T_{p}d^{k}10^{\alpha (f)d/10}$$

where *P*_0_ denotes the power threshold for a node to receive the information package. *T_p_* denotes transmission delay of the information package, *d* denotes the transmitting distance of the information package, and *k* is the energy spreading factor. The absorption coefficient *α(f)* can be calculated through the following formula:

(4) $$\alpha (f)=0.11\frac{f^{2}}{1+f^{2}}+44\frac{f^{2}}{4100+f^{2}}+2.75\times 10^{-4}f^{2}+0.003$$

where *f* is the frequency of the carrier acoustic signal in *HZ*, and *α(f)* is in *dB/m*. In addition, the movement energy consumption *M_e_* of node can be expressed as the product of the movement distance *m_d_* and the energy consumption of per movement distance is *m_u_*. The relationship is described as follows:

(5) $$M_{e}=m_{d}*m_{u}$$

### 3.2. Related Definitions

#### 3.2.1. Network Coverage Rate

**Figure 2:**
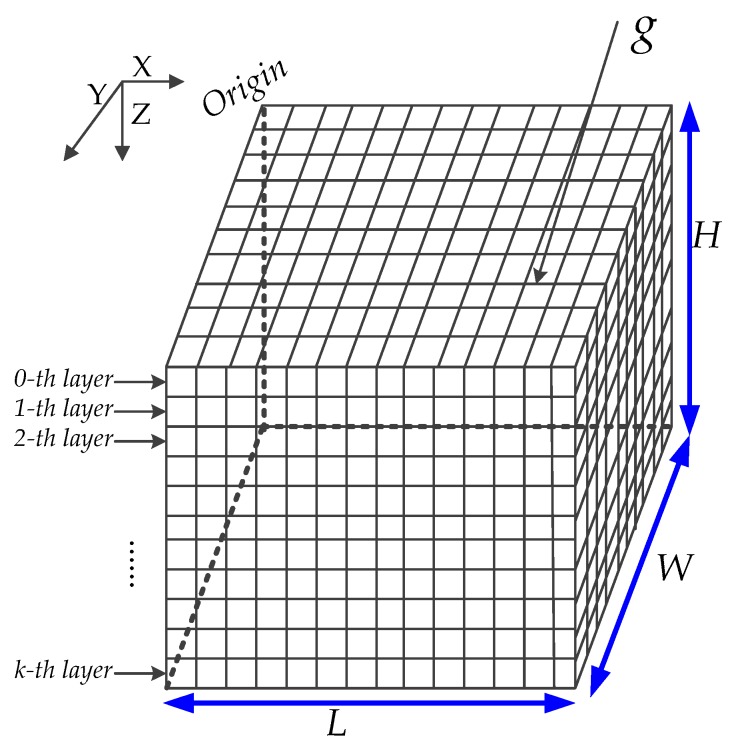
Grid division for 3D UWSNs.

As shown in Figure 2, the 3D UWSNs are divided into a plurality of small cube grid *g*, and all of small grids position can be presented by selecting the coordinates of the center of these grids. Through the Boolean perception model (Section 3.1.2), the network coverage rate *C_c_* is defined by the ratio of the number *N_S_* of grid centers covered in the monitored underwater environment space to the total number *N_N_* of grids:

(6) $$C_{c}=\frac{N_{S}}{N_{N}}$$

#### 3.2.2. Network Connectivity Rate

Network connectivity rate *C_n_* is defined as the ratio of *n_c_* to *n*, where *n_c_* denotes the number of nodes that can communicate with the sink node through sing-hop or multi-hop communication, and *n* is the total number of nodes in the monitored underwater environment space, therefore, *C_n_* can be calculated as follows:

(7) $$C_{n}=\frac{n_{c}}{n}$$

If *C_n_* is equal 1, the network achieves full connectivity, namely, all nodes can communicate with the sink node through single-hop or multi-hop communication.

#### 3.2.3. Network Reliability

As a crucial indicator for evaluating the network quality of service (QoS) [32], network reliability is the probability that the network can maintain full network connectivity at a high level. This study mainly investigates UWSNs deployment issues. Thus the network should still maintain a high network connectivity rate, which is a crucial optimization goal of our algorithm, as some nodes begin to die because of persistent network operation. Therefore, in this study, network reliability can be described as the average node degree (not in the same depth):

(8) $$D_{a}=\frac{\sum_{i=1}^{n}N_{i}(nei)}{n}$$

where *D_a_* denotes the average node degree, *N_i_*(*nei*) denotes the neighbor node number of node *S_i_*.

#### 3.2.4. Two-Dimensional Convex Hull

Given a points set *p =* {*p_t_, t =* 1, 2, …, *n*} on a 2D plane *V*, any finite points at set *p* constructs all of convex combination and then forms a set *H*, which is the convex hull of set *p* and is denoted by *H*(*p*) [33]:

(9) $$H(p)=\left\{\sum_{i=1}^{m}\varepsilon_{i}x_{i}|x_{i}\in p,\varepsilon_{i}\geq 0,i=1,2,...,m,\sum_{i=1}^{m}\varepsilon_{i}=1,m\in N\right\}$$

**Figure 3:**
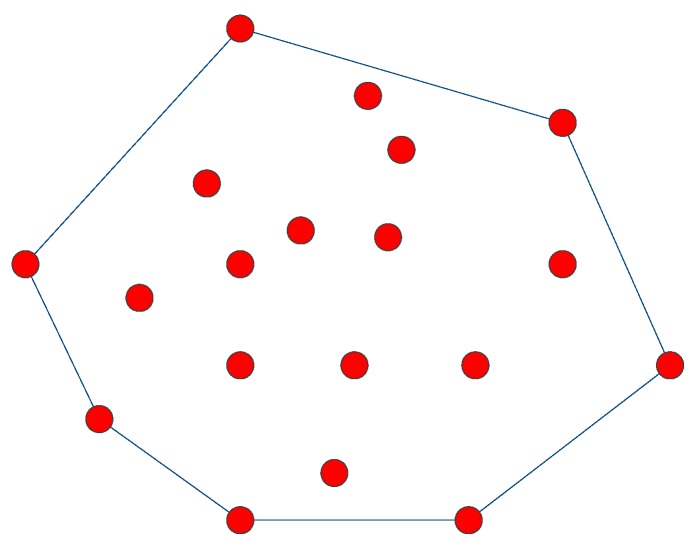
Schematic of a 2D convex hull.

Concretely, *h* is the minimum convex polygon in the 2D plane *V* that can enclose all points of set *p* and is referred to as the 2D convex hull of the set *p*. As shown in Figure 3, the geometry is a convex hull of the point set surrounded by solid lines.

#### 3.2.5. Empty Circle

A point set *p =* {*p_t_, t =* 1, 2, …, *n*} is given on a 2D plane *V*. A circle *C_m_* (*p_i_*, *p_j_*, *p_k_*) is constructed with three out-of-line points *p_i_*, *p_j_*, *p_k_*, and this circular region does not contain any arbitrary point within set *p.* Moreover, the center of circle *C_m_* is located in given plane *V* and is an empty circle.

## 4. Problem Analysis and Algorithm Description

### 4.1. Problem Analysis

In using energy-constrained UWSNs to monitor a target water area, we must ensure that the network has high coverage and full connectivity at the same time and effectively manage nodes sleep or active states to save communication energy consumption. Moreover, the network should maintain high network connectivity, which is a crucial optimization goal of our algorithm, as some nodes begin to die because of persistent network operation, that is, the network still has relatively good reliability. However, in most of the move-constrained deployment algorithms of [13,14,15], coverage overlaps are reduced by node depth adjustment, thus increasing the global network coverage rate. On the other hand, all nodes are in an active state during the deployment process, and this increases their network communication energy use. Meanwhile, these algorithms do not consider the average node degrees between different depth networks, subsequently affecting network reliability and connectivity rate under network operation conditions. Moreover, in view of the node move-restricted depth-adjustment algorithm based on computational geometry, through the vertical depth-adjustment of nodes in reducing network coverage redundancy, the 3D network coverage is increased, Wu et al. [24] proposed the VBDA algorithm for UWSNs. However, the VBDA algorithm does not consider the network connectivity between two neighboring layers, resulting in a very poor network connectivity performance with a small *α* (the ratio of node communication radius to sensing radius). As the network is deployed, fewer nodes are available to execute depth adjustment when the process of deployment reaches the water bottom threshold. Thereafter, network coverage performance becomes relatively inferior. In addition, the VBDA algorithm does not consider network connectivity in the deployment process. The nodes are always in the active state, and thus all nodes require more communication, thereby consuming more communication energy. The limited energy for the nodes and the difficulty of charging the batteries in the water monitoring space affect the network operator performance, including network coverage rate, network connectivity rate, and network reliability. Meanwhile, VBDA is a centralized algorithm, and its practical application is more difficult to achieve than that of our proposed system.

To better solve the problem of the abovementioned VBDA algorithm, we proposed NDACS in this study. A detailed description of the NDACS algorithm is given in the next section.

### 4.2. Algorithm Description

**Figure 4:**
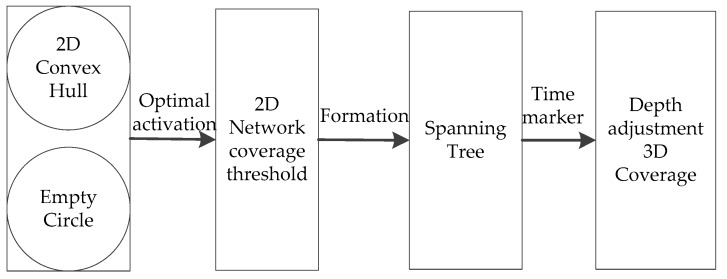
Algorithm framework.

The main ideas of the NDACS algorithm can be described as the following steps: (1) optimal activation strategy based on 2D convex hull and empty circle on the water surface; (2) formation process of a 2D convex hull spanning tree; (3) and depth-adjustment strategy based on time markers in achieving 3D network coverage. The algorithm framework is shown in Figure 4.

#### 4.2.1. Optimal Activation Strategy Based on 2D Convex Hull

**Figure 5:**
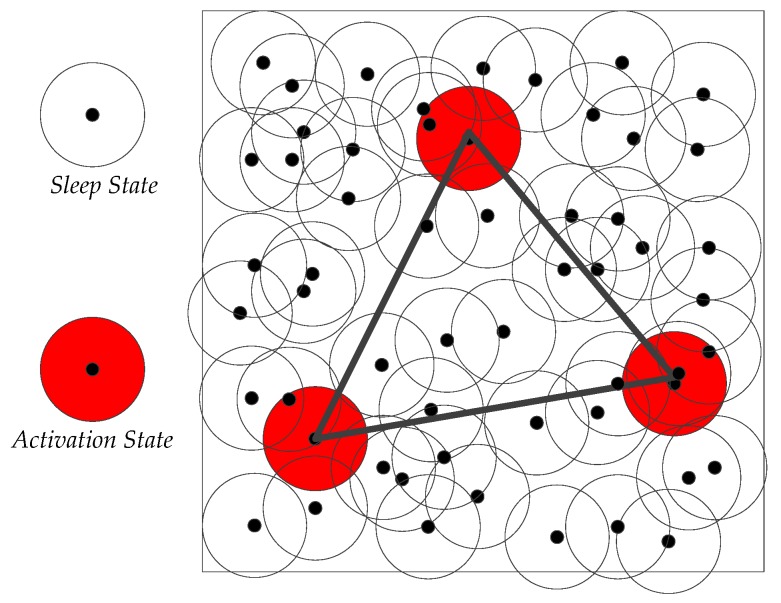
Schematic diagram of the 2D convex hull activation strategy.

In minimizing network coverage overlaps, nodes should be as far away from one another as possible, but they should not interrupt the communication links between source nodes and their neighboring nodes. The sink node broadcasts a message to all nodes, according to the received signal strength from node *S_i_*, to calculate the distance between *S_i_* and the sink node. In a similar manner, the 2D level coordinates of all nodes can be determined based on the sink node. The sink node sends a control signal to randomly activate three sleep nodes, which then form the simplest 2D convex hull, as shown in Figure 5. In the figure, the active nodes form a simplest 2D convex hull (a triangle) through a solid line. The set of currently active nodes is denoted by Sa, and the set of sleep nodes is denoted by *Ss*. Sleep nodes inside the 2D convex hull are referred to as *sleep_in*, and those outside are denoted by *sleep_out*, where *sleep_in ∩ sleep_out = Ss.*

The inside and outside of the 2D convex hull have many coverage holes and monitoring blanks. Obviously, the current regional network coverage area is determined by two parts: the coverage holes of the convex hull interior and the monitoring blanks of the convex hull exterior. Therefore, we obtain the following formula:

(10) $$S_{c}=S_{CH}-\;S_{Hole}$$

where *S_c_* denotes the 2D network coverage area, *S_CH_* denotes the area of the 2D convex hull, and *S_Hole_* denotes the area of the coverage holes. Thus, we can improve the value of *S_c_* by increasing *S_CH_* or decreasing *S_Hole_*.

Furthermore, the coverage area of the network can be expressed:

(11) $$S_{c}=\overset{m}{\underset{i=1}{\cup }}C_{i}$$

where *C_i_* denotes the coverage area of the active node *S_i_*. *m* nodes are activated and $S_{i}\in Sa$.

A sleep node *C_j_* is chosen from *Ss* to be activated, and then the area of the 2D network coverage is maximized. At the current moment, the coverage area is presented by $S_{c}^{'}$, and our goal is to maximize $S_{c}^{'}$.

(12) $$\max{S_{c}}^{'}=S_{c}\cup C_{j}=\left(\overset{m}{\underset{i=1}{\cup }}C_{i}\right)\cup C_{j}$$

where *C_j_* denotes the coverage area of sleep node *S_j_*, and $S_{j}\in Ss$.

To maximize $S_{c}^{'}$, by comparing the different coverage gains from selecting the different activated sleep nodes derived from *sleep_in* or *sleep_out*, we determine the node that can obtain the greatest coverage gains. Therefore, the optimal activation strategy is transformed into the maximum coverage gains through the selection of activated sleep nodes in *Ss*. Before the optimal activation strategy is solved, the following theorem is proposed, and we will prove it subsequently.

**Theorem** **1.**  If the radius Rh of sn empty circle (Section 3.2.5) is greater than the sensing radius Rs, then the interior of the empty circle will certainly have coverage holes.

**Proof of Theorem** **1.**  By the definition in Section 3.2.5, the interior of the empty circle must not have additional active nodes, which means that the interior of the empty circle maybe has some sleeping nodes. Obviously, when *Rh* is greater than *Rs*, the center of the empty circle must not be covered, so we can infer that coverage holes exist in the empty circle.

When *Rh* is larger, the active nodes are relatively more sparse, and the probability of occurrence of coverage holes is greater. Therefore, we choose to activate a sleeping node from the interior of the empty circle with a maximum *Rh*, and this activation strategy can effectively increase the network coverage area. At this moment, in selecting to activate the sleep node nearest to the center of this empty circle, the node becomes a candidate active node and is denoted by *S_Cand_*_1_.

Moreover, the activation of a sleeping node from the exterior of the 2D convex hull can increase the area of the convex hull, thus forming a new 2D convex hull. In selecting to activate a sleeping node that can maximize the area of convex hull, the node becomes a candidate active node denoted by *S_Cand_*_2_.

The next optimal active node must be either *S_Cand_*_1_ or *S_Cand_*_2_, and the optimal activation strategy is shown in Formula (13):

(13) $$Opt=\left\{ \begin{array}{l} S_{Cand1},\;E\_circle\geq H\_add\\ S_{Cand2},\;H\_add<E\_circle \end{array}\right.$$

where *E_circle* denotes the maximum area of the empty circle when the active node is *S_Cand_*_1_, *H_add* denotes the maximum area increase of the 2D convex hull when the active node is *S_Cand_*_2_, and *Opt* denotes the node selected to be activated ultimately.

**Figure 6:**
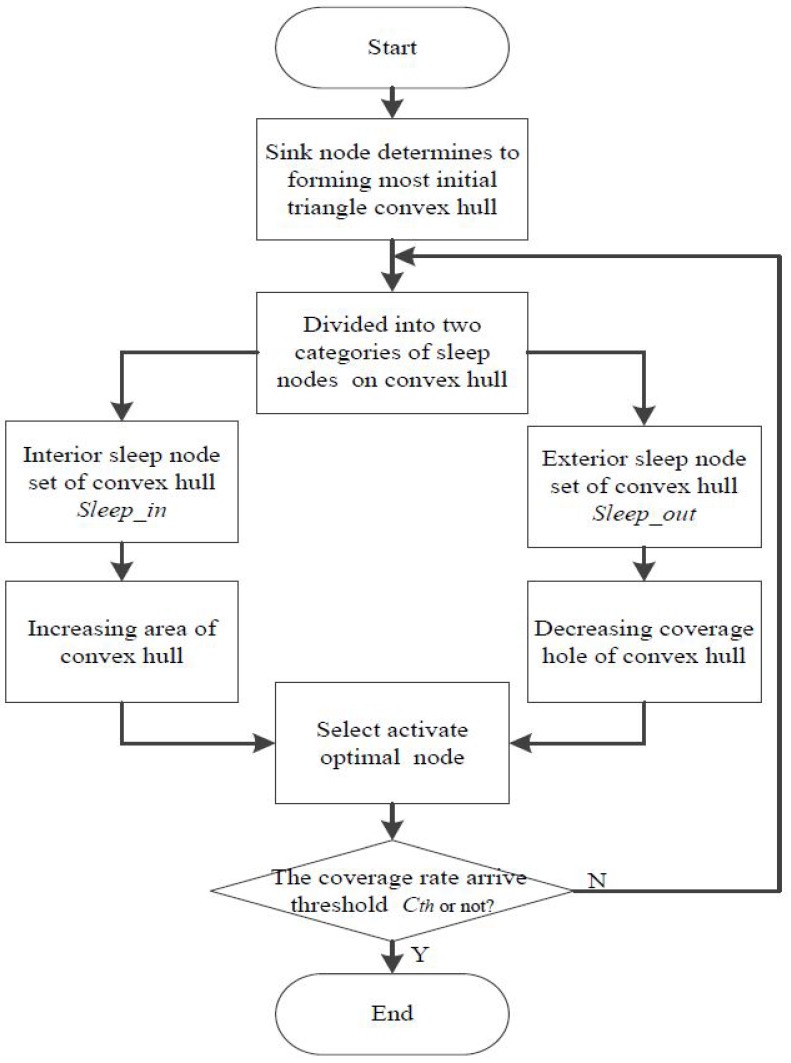
Flowchart of the optimal activation strategy.

Node *Opt* will be added to *Sa* and deleted from *Ss*. Thereafter, in determining whether the current network coverage rate would satisfy the coverage requirement, if the result does not satisfy the requirement, then the abovementioned strategy is adopted to continue activating a new sleeping node until the coverage threshold *C_th_* is reached in this horizontal plane. Thus the whole optimal activation strategy process is completed, and the detailed flowchart of the activation strategy is as shown in Figure 6.

#### 4.2.2. Formation Process of 2D Convex Hull Spanning Tree

The sink node is the tree root node *r* found at the center of the water surface. It broadcasts information to other active nodes, according to the received signal strength from node *S_i_*, to calculate distance *ds_i_* (1 ≤ *i* ≤ *N_A_*) between *S_i_* and the sink node, and *N_A_* denotes the number of active nodes. Moreover, the sink node assigns a unique activation identifier *ID_A_* to all active nodes, where *ID_A_* is recorded from 1 to *N_A_* according to the size of *ds_i_*. Furthermore, all active nodes in the convex hull are recorded as *CH_nodes*.

**Figure 7:**
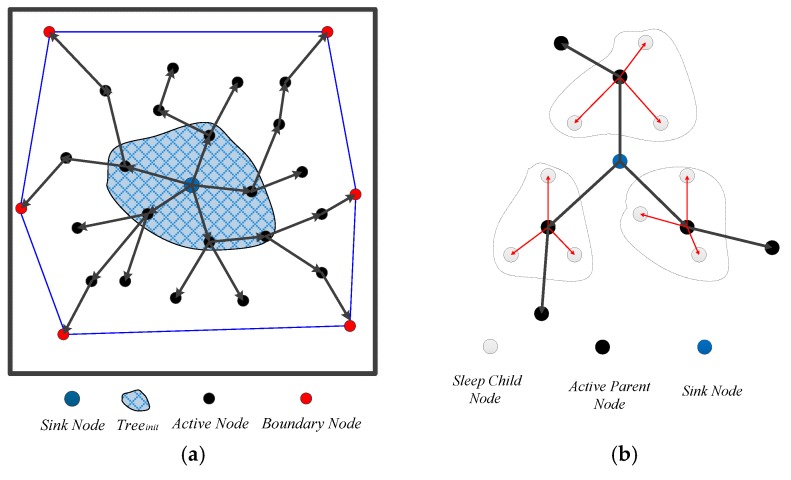
(**a**) Initial spanning forest and (**b**) Spanning tree consisting of hybrid construction.

(1) The tree root node *r* and set of active neighbor nodes *Nei_A* form an initial spanning tree *Tree_init_* as shown in Figure 7a. The iterative strategy is adopted to produce a next spanning tree generation according to the size of *ID_A_*, until all active nodes of the 2D convex hull form a complete initial spanning forest *Forest_init_*. Whether a parent node and its child nodes on spanning forest *Forest_init_* are able to communicate, if not, by Section 3.1.3, the communication radius is incremented until the parent node is connected to its child nodes so that the initial spanning forest *Forest_init_* can maintain full network connectivity.
(2) The time marker *T_mark_* $\left(0\leq T_{mark}\leq T\right)$of root node *r* is initialized to 0, and the next generation of child nodes’ time marker increments by 1, until all active nodes of the initial spanning forest *Forest_init_* are marked to the *T* level (i.e., all of *CH_node* are marked by the *r* node). The *T_mark_* of the same subtree parent node set {*f_j_^i^*} (*j* denotes *ID_A_*; *i* denotes *T_mark_*) is recorded.
(3) The active node inside the 2D convex hull *Sa*_in (*ID_A_*) acts as the parent of its neighboring sleeping nodes. Building a small spanning tree of individual nodes on *Forest_init_*, we define a small spanning tree consisting of hybrid construction where the parent is the active node and the child is the sleeping node, as shown in Figure 7b.
(4) To achieve the 3D global network coverage, the algorithm needs to deploy neighboring sleeping nodes *Nei*(*f_j_^i^*) distributed at different depths and calculate the depth adjustment of the set of subtree parent nodes {*f_j_^i^*} for their child sleeping nodes.

#### 4.2.3. Depth-Adjustment Strategy Based on Time Markers

**Figure 8:**
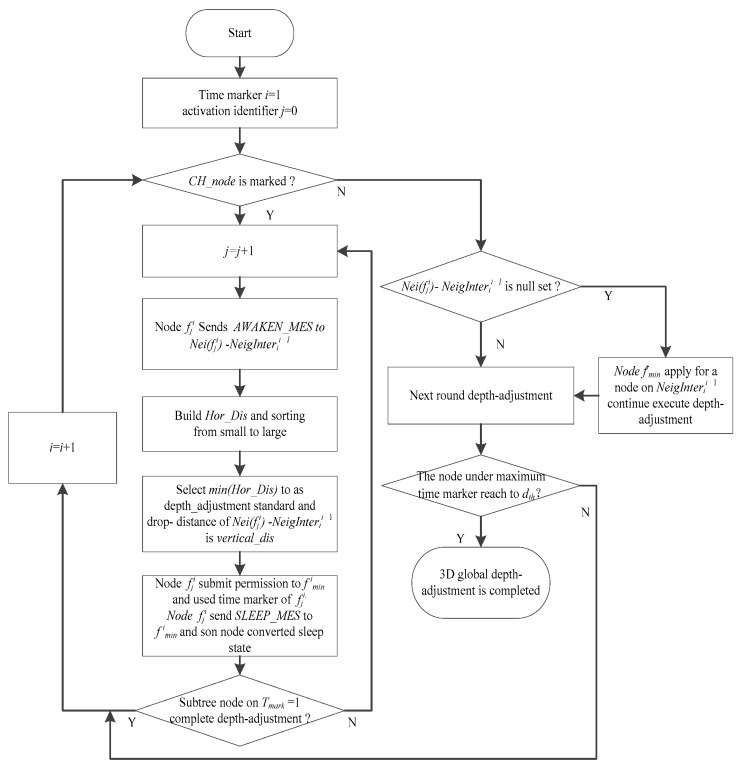
Flowchart of the depth-adjustment strategy.

For different parent nodes in {*f_j_^i^*}, we use a time marker *T_mark_* to define network hierarchy. We need to consider the network energy consumption balance. A node near the sink node has low network hierarchy and high probability of data transmission and relay so it needs more energy. Therefore, for the network to have a sustained and stable operation, we should attempt to deploy more nodes in near the sink node and ensure that the surrounding sink node contains more subtrees. Moreover, subtree size should be as small as possible. Our goal is to reducing the redundancy degree of network coverage, increase the 3D space network coverage rate, and maintain constant full network connectivity between subtree parents and child nodes. This study proposes a strategy is based on the depth-adjustment strategy of time markers, and its detailed description is as follows:

(1) The set of neighbor nodes {*Nei* (f_*_^i^)} under the same network hierarchy (the same time marker *i*) are recorded, and then whether two sets of neighboring sleeping nodes intersect under the neighbor time marker and same path of tree is determined. If an intersection exists, then it is *NeigInter_i_^+^*^1^ between time marker *i* and *i* + 1; otherwise, an empty set is recorded to *NeigInter_i_^i+^*^1^.
(2) Time marker *T_mark_* is initialized to 1, that is, *T_mark_* = 1. The active node *f_j_^i^* inside the convex hull sends an awaken message *AWAKEN_MES* to the set of non-intersecting neighboring nodes *Nei(f_j_^i^) − NeigInter_i_^i+^*^1^ and activates them. Node *f_j_^i^* builds a horizontal distance state table *Horizontal_Dis*(*f_j_^i^*, *Nei(f_j_^i^) − NeigInter_i_* ^*i*+1^) according to the distance between the child elements of *Nei(f_j_^i^) − NeigInter_i_^i+^*^1^ and their parent nodes *f_j_^i^* from small to large. Thereafter, a node *f^i^_min_* is selected with a minimum value *min*(*Hor_Dis*) on the state table as a depth-adjustment standard. Therefore, the drop-distance *ver_dis* of non-intersecting neighbor nodes set *Neig(f_j_^i^) − NeigInter_i_^i+^*^1^ is calculated by Equation (14):
  (14) $$ver\_dis=\sqrt{R{c_{\max}}^{2}-{\left\{min\left(Hor\_Dis\right)\right\}}^{2}}$$
  where *Rc_max_* denotes the maximum communication radius after adjusted and can maintain connectivity between parent node *f_j_^i^* and its child node.
(3) Furthermore, node *f_j_^i^* submits a permission to its child node *f^i^_min_* and makes it as a parent node of the set of neighbor nodes *Nei*(*f_j_^i^*) − *NeigInter_i_^i+^*^1^. Thereafter, *f^i^_min_* is still used its time marker of parent node *f_j_^i^*. According to the size of the order of *ID_A_* to all neighbor nodes on the same time marker adopting the depth-adjustment method of step (2) to adjust the depth of the other child nodes of *Nei*(*f_j_^i^*) − *NeigInter_i_^i+^*^1^, node *f_j_^i^* sends a sleep message *SLEEP_MES* to its neighbor node and converts them to sleep state. Thereafter, *T_mark_* = *T_mark_* + 1, and the above depth-adjustment process is repeated for *f_j_^i^* until all *CH_node* is marked, achieving a layer depth adjustment.
(4) The horizontal distance state table *Hor_Dis*(*f_j_^i^*, *Nei*(*f_j_^i^*) − *NeigInter_i_^i+^*^1^) is updated, and node *f^i^_min_* is adopted according to Steps (2) and (3) to achieve the next layer of the depth adjustment of its child node continually. If the node in this time marker drops down to a depth threshold *d_th_*, then the depth adjustment on this time marker is completed. If the process finds no node to execute depth adjustment, that is, the set of neighbor nodes *Nei*(*f_j_^i^*) − *NeigInter_i_^i+^*^1^ is a null set, then node *f^i^_min_* applies to request other nodes in the set of neighbor intersections *NeigInter_i_^i+^*^1^ to continue the process of depth adjustment until all child nodes of the entire time marker complete the whole depth-adjustment process, finishing the global 3D network coverage deployment. The flowchart of the depth-adjustment strategy is shown in Figure 8.

## 5. Complexity Analysis

### 5.1. Communication Complexity Analysis

After the sink node completes the 2D convex hull activation process, the communication complexity of the process can be obtained, and it is *O*(*n × h*) according to the *GiftWrapping* algorithm [34], where *h* denotes the required number of active nodes to achieve the 2D convex hull, and its asymptotic value is ${L*W/\pi *Rs}^{2}$. Given that the numbers of elements in *Nei(f_j_^i^) − NeigInter_i_^i+^*^1^ and *NeigInter_i_^i+^*^1^ are *N_ij_* and $N_{c}^{i}$ respectively, node *f_j_^i^* broadcasts a message to its neighbor child nodes, and all parent nodes compare the distances of all neighbor child nodes and must send a total of h*(n−h) messages for an overall situation significance. Node *f_j_^i^* submits the permission to its child node *f^i^_min_* for *k* times through an iterative method, sending a total of *k × N_A_* messages. The number of messages that must be sent by the parent node is twice the total number of nodes in the 2D convex hull activation process, namely, 2*n*. After network deployment is completed, the value of *N_A_* and *n* are approximately equal. Therefore, the complexity of network communication is O(*h ×* (*n − h*)) *+* O(*k × N_A_*) *+* O(*2n*), then the big-O complexity is O(*cn*), where *c = k + h + 2*.

### 5.2. Time Complexity Analysis of Network Deployment

Compared with TWSN deployment algorithms, UWSN deployment algorithms include the time cost of depth adjustment and the acoustic transmission delay. During network pre-deployment, the sink node calculates the optimal activation node position, and because the sink node’s hardware configuration is high and has ability of fast calculation speed in general conditions, its time cost in the convex hull activation process can be ignored. The time cost is *T_c_* of the node state changing from sleep to active or inversely, and acoustic transmission delay is *T_d_*. Therefore, the total communication time cost is 2(*Rc/p + T_d_*) + *T_c_* between parent and its child nodes in the process of the spanning tree algorithm. Node *f_j_^i^* submits the permission to its child nodes *f^i^_min_* repeatedly, and its total time cost is *k × Rc/p* + *k ×* (2*T_d_ + T_c_*). After the 2D spanning tree process is completed, approximately (*n − h*) child nodes need to execute depth adjustment, and they will spend *H/v* until the water bottom in the worst case. Therefore, the average time cost of the global network deployment is around $2\left(Rc/p+T_{d}\right)+T_{c}+k*Rc/p+k*(2T_{d}+T_{c})+H/v$, and the big-O time complexity of the NDACS algorithm is $O\left(H/v+k*Rc/p+k*(T_{d}+T_{c})\right)$. And the related symbolic descriptions are defined in Table 1.

## 6. Simulation Evaluation

### 6.1. Parameter Settings and Evaluation Metrics

In analyzing and verifying the effectiveness of the NDACS algorithm, it is compared with VBDA in this study. Because VBDA is a node depth-adjustment deployment algorithm based on computational geometry, nodes can only move along the vertical direction, and VBDA’s various hypotheses and preliminary conditions are similar to those of the NDACS algorithm. The algorithms are compared according to network connectivity rate and network coverage rate, energy consumption of communication and movement, and network reliability in this study.

MATLAB is used to simulate and analyze experiment results, and the simulation result is the mean of 30 experiments. In verifying the feasibility of the results, the 3D volume of water monitored is 500 m × 500 m × 400 m; the sensing radius *Rs* of the node is 50 m; the initial communication radius *Rc* is 60 m; and *Bu* is 5 m. When the network is deployed, the network coverage rate and connectivity rate are compared. Energy consumptions are compared based on the total energy consumed during the deployment process, and the rest of the parameters are set as shown in Table 2, with other settings derived from [30].

### 6.2. Comparison and Analysis of Simulation Results

#### 6.2.1. Network Coverage Rate

**Figure 9:**
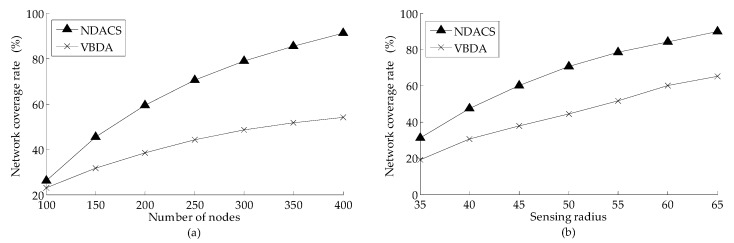
Comparison of network coverage rate between NDACS and VBDA: (**a**) comparison of network coverage rate between NDACS and VBDA varying with the number of nodes, and node sensing radius is 50 m; (**b**) comparison of network coverage rate between NDACS and VBDA varying with sensing radius, and the default number of nodes is 200.

Figure 9 shows the comparison of the network coverage rate that varies with the number of nodes and the sensing radius on the NDACS and VBDA, respectively. Figure 9a shows the comparison of the network coverage rate varying with the number of nodes on the NDACS and VBDA, where the default sensing radius is 50 m in the simulation scenario. Figure 9b shows the comparison of the network coverage rate that varies with the sensing radius of the NDACS and VBDA, where the default number of nodes is 200 in the simulation scenario. As shown in Figure 9a, compared with the VBDA, the NDACS algorithm can maintain a higher network coverage rate consistently under the same sensing radius because VBDA can only guarantee the upper layer network with high network coverage but does not consider the coverage overlaps between its neighbor interlayer, resulting in the failure of deeper waters to achieve better coverage. Figure 9b shows that with the increase sensing radius, the NDACS algorithm for network coverage rate increases steadily and consistently outperforms VBDA. This is mainly because the NDACS algorithm, after completing the 2D convex hull activation process, executes depth-adjustment strategy of the time marker to greatly reduce the network coverage overlaps, and the set *NeigInter_i_^i+^*^1^ is established to provide more adjustment candidates on deeper location network deployment. Therefore, the NDACS algorithm can greatly improve the network coverage rate.

#### 6.2.2. Network Connectivity Rate

**Figure 10:**
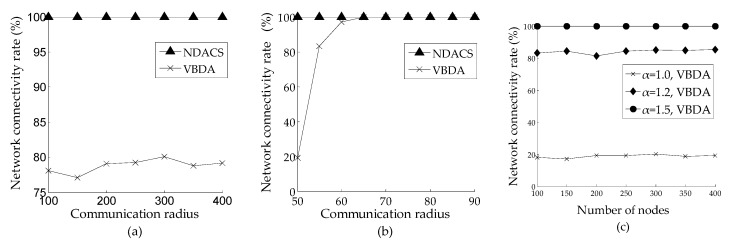
Comparison of network connectivity rate between NDACS and VBDA: (**a**) comparison of network connectivity rate between NDACS and VBDA varying with number of nodes, and the initial communication radius is 60 m; (**b**) comparison of network connectivity rate between NDACS and VBDA varying with communication radius, and the default number of nodes is 200; (**c**) network connectivity rate of VBDA varying with number of nodes on different values of α.

Figure 10 shows the comparison of the network connectivity rate that varies with number of nodes and the communication radius on NDACS and VBDA, respectively. Figure 10a shows the comparison of the network connectivity rate varying with the number of nodes on NDACS and VBDA, where the default initial communication radius is 60 m in the simulation scenario. Figure 10b shows the comparison of the network connectivity rate varying with the communication radius of NDACS and VBDA, where the default number of nodes is 200 in the simulation scenario. Figure 10a,b show that compared with VBDA, the NDACS algorithm can guarantee the full network connectivity of the network (i.e., the network connectivity rate is equal 1). Figure 10c shows that VBDA can only maintain a relatively high network connectivity rate when α > 1.2 (*α* is the ratio of node communication radius to sensing radius), and its connectivity rate will be very small in other situations. However, For the NDACS algorithm, the first layer network can guarantee full network connectivity on the spanning tree. In the depth-adjustment process, the parent node minimizes network coverage overlaps and maintains full network connectivity by using the adjustable communication radius model described in Section 3.1.3.

#### 6.2.3. Energy Consumption of Communication and Movement

**Figure 11:**
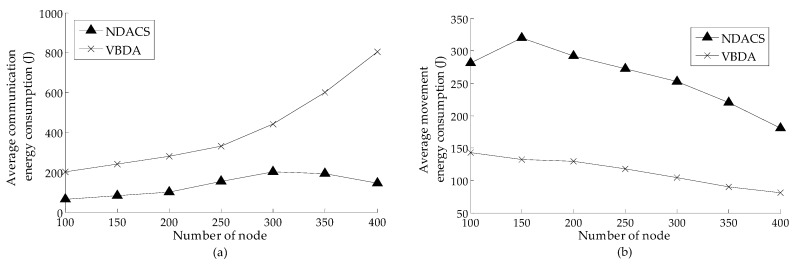
Comparison of energy consumption between NDACS and VBDA: (**a**) comparison of node average energy consumption of communication between NDACS and VBDA varying with the number of nodes, and the initial communication radius is 60 m; (**b**) comparison of node average energy consumption of movement between NDACS and VBDA varying with the number of nodes.

Figure 11a,b show the comparison of the each node’s average energy consumption of communication and movement varying with the number of nodes on NDACS and VBDA, respectively, where the value of initial communication radius is 60 m in the simulation scenario (default settings for other parameters). As shown in Figure 11a, for VBDA, its rate of increase of average communication energy consumption is relatively fast as the number of nodes increases, and its value is always greater than that of the NDACS algorithm. This is because VBDA needs more communication energy, and all nodes are in an active state and must broadcast messages to their surrounding neighbor nodes. However, since the NDACS algorithm only needs some nodes in the active state, their states can be transformed flexibly through the spanning tree strategy. Therefore, VBDA needs more communication energy when the number of nodes increases. As shown in Figure 11b, the average movement energy consumption of VBDA presents a decreasing trend with the increase of the number of nodes. However, the average movement energy consumption of the NDACS algorithm surpasses that of VBDA, and it initially presents an increasing then decreasing trend. With this finding, the NDACS only executes depth-adjustment strategy on the set of non-intersecting neighbor nodes *Nei*(*f_j_^i^*) − *NeigInter_i_^i+^*^1^ for a long period of time. However, when *Nei(f_j_^i^)* − *NeigInter_i_^i+^*^1^ is null set, the NDACS algorithm may require the set *NeigInter_i_^i+^*^1^ to execute depth adjustment and deploy them to a deeper location such that they will consume more movement energy. Overall, as shown in Figure 10a,b, the total average energy consumption of VBDA is greater than that of the NDACS.

#### 6.2.4. Network Reliability

**Figure 12:**
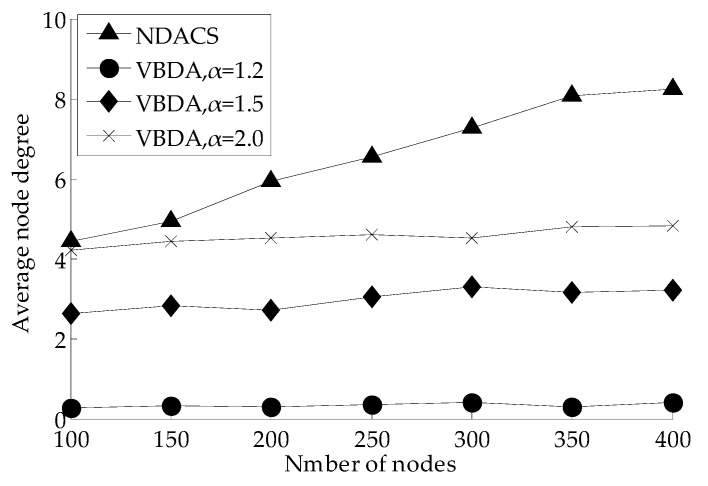
Comparison of average node degree between NDACS and VBDA.

Network reliability is defined in Section 3.2.3 as a criterion of QoS for UWSNs. In this section, Figure 12 shows the comparison of the average node degree varying with the number of nodes on NDACS and VBDA, and the influences of the simulation results on the different values of parameter α to average node degree are analyzed. As shown in Figure 12, compared with VBDA, the NDACS algorithm can maintain a higher average node degree, but the growth of the average node degree of VBDA is gentle, with an increment of the total nodes’ number. With this finding, the NDACS algorithm can adjust the communication radius by using the adjustable communication radius model in Section 3.1.3 and considering real time connectivity between the parent node and its child nodes. However, VBDA cannot completely guarantee interlayer connectivity under different values of parameter α (as shown in Figure 10c. Therefore, the NDACS algorithm has a higher average node degree, which can maintain stronger communication connectivity between the node and the base station on the water surface, thus network operation is more reliable.

## 7. Conclusions

This study addressed the problems of node depth-adjustment deployment (vertical movement deployment) for UWSNs. We have proposed NDACS, that forms a connected 2D convex hull spanning tree first and then connects the nodes of a spanning tree to calculate the depth-adjustment distances of their neighbor nodes based on time marker while maintaining the connectivity between the parent node and their neighbor child nodes through the introduction of the mechanism of adjustable communication radius model, until the node deploys to the depth threshold and achieves the overall network coverage deployment. Through simulation experiments, we have evaluated the different indicators including network connectivity rate and network coverage rate, energy consumption of communication and movement, and network stability. The comparison of these simulation results shows that the various performances of the NDACS algorithm are better than those of VBDA. The symbolic notation of the NDACS algorithm is described in Table 3.

In the future, we plan to study the efficiency of network data acquisition when achieving network deployment by designing a more proper routing strategy for this deployment algorithm scenario. In real-world network systems, usually the model of communication and sensing is based on probability. The probability model of communication and sensing is worthwhile to adopt on simulation setup, then executes comparative analysis on experiment results. Meanwhile, the redeployment of the set of nodes *NeigInter_i_^i+^*^1^ is also worthy of further study. Furthermore, adding the obstacle model or water mobility model to increase the practical applicability of the algorithm is also included in our future research work.

## Figures and Tables

**Table 1:** Symbolic description of complexity analysis.

H	Depth of Monitoring Space
v	Movement speed of node
K	Number of times of depth-adjustment
Rc	Communication radius of node
P	Speed of sound
T_d_	Propagation delay of acoustic signal
T_c_	Transition time from active to sleep or inversely

**Table 2:** Settings of Simulation Parameters.

Parameter	Value
Initial energy of node *E_init_*	16,000 J
Coverage threshold *C_th_*	0.9
Energy consumption on unit moving distance *m_u_*	2 J/m
Power threshold *P*_0_	0.05 W
Transmission delay *T_p_*	0.2 s
Energy spreading factor *k*	2
Carrier frequency *f*	24 kHz
Sense radius of node *Rs*	50 m

**Table 3:** Main Symbolic Notation of the NDACS Algorithm.

Symbol	Definition
*N*	Number of nodes
*ID*	Node unique identifier
*Rs*	Node sensing radius
*Rc*	Node communication radius
*Rc_init_*	Initial communication radius
θ	Adjustable level of communication radius
*B_u_*	Accumulation of communication radius
*S_i_*	ID is *i* of node
*D*	Transmitting distance of the information package
*P*_0_	Power threshold of packets can be received
*F*	Carrier frequency
*T_p_*	Transmission delay of data transmission
*K*	Energy spreading factor
*M_e_*	Movement energy consumption of node
*m_d_*	Node movement distance
*m_u_*	Energy consumption on unit moving distance
*Ss*	Set of sleep nodes
*Sa*	Set of active nodes
*sleep_in*	Set of sleep nodes inside the convex hull
*sleep_out*	Set of sleep nodes outside the convex hull
*S_c_*	Network coverage area on process of convex hull activation
*S_CH_*	Area of 2d convex hull
*S_Hole_*	Area of coverage holes
*Rh*	Empty circle radius
*N_A_*	Total number of active nodes
*Nei_A*	Active neighbor nodes set
*Nei*(*f_j_^i^)*	Neighbor nodes set of node *f_j_^i^*
*NeigInter_i_^i+^* ^1^	Insertion of neighbor sleep nodes
{*f_j_^i^*}	Parent node set of subtree
*T_mark_*	Time marker of active node
*d_th_*	Depth-adjustment threshold of network
*C_th_*	Coverage threshold on 2d network surface
*ID_A_*	Activation identifier of node
*C_c_*	Network coverage rate
*C_n_*	Network connectivity rate
*Α*	Ratio of node communication radius to sensing radius
